# In Vitro Study of the Toxicity Mechanisms of Nanoscale Zero-Valent Iron (nZVI) and Released Iron Ions Using Earthworm Cells

**DOI:** 10.3390/nano10112189

**Published:** 2020-11-03

**Authors:** Jaroslav Semerad, Natividad Isabel Navarro Pacheco, Alena Grasserova, Petra Prochazkova, Martin Pivokonsky, Lenka Pivokonska, Tomas Cajthaml

**Affiliations:** 1 Institute of Microbiology of the Czech Academy of Sciences, Vídeňská 1083, CZ-142 20, Prague 4, Czech Republic; jaroslav.semerad@biomed.cas.cz (J.S.); natividad.pacheco@biomed.cas.cz (N.I.N.P.); alena.grasserova@biomed.cas.cz (A.G.); kohler@biomed.cas.cz (P.P.); 2 Institute for Environmental Studies, Faculty of Science, Charles University, Benátská 2, CZ-128 01, Prague 2, Czech Republic; 3 First Faculty of Medicine, Charles University, Kateřinská 1660/32, CZ-121 08, Prague 2, Czech Republic; 4 Institute of Hydrodynamics of the Czech Academy of Sciences, Pod Paťankou 30/5, CZ-166 12, Prague 6, Czech Republic; pivo@ih.cas.cz (M.P.); pivokonska@ih.cas.cz (L.P.)

**Keywords:** nanoecotoxicology, earthworms, coelomocytes, reactive oxygen species, nanoscale zero-valent iron (nZVI), ferrous and ferric ions, phagocytosis, lipid peroxidation, apoptosis

## Abstract

During the last two decades, nanomaterials based on nanoscale zero-valent iron (nZVI) have ranked among the most utilized remediation technologies for soil and groundwater cleanup. The high reduction capacity of elemental iron (Fe^0^) allows for the rapid and cost-efficient degradation or transformation of many organic and inorganic pollutants. Although worldwide real and pilot applications show promising results, the effects of nZVI on exposed living organisms are still not well explored. The majority of the recent studies examined toxicity to microbes and to a lesser extent to other organisms that could also be exposed to nZVI via nanoremediation applications. In this work, a novel approach using amoebocytes, the immune effector cells of the earthworm *Eisenia andrei*, was applied to study the toxicity mechanisms of nZVI. The toxicity of the dissolved iron released during exposure was studied to evaluate the effect of nZVI aging with regard to toxicity and to assess the true environmental risks. The impact of nZVI and associated iron ions was studied in vitro on the subcellular level using different toxicological approaches, such as short-term immunological responses and oxidative stress. The results revealed an increase in reactive oxygen species production following nZVI exposure, as well as a dose-dependent increase in lipid peroxidation. Programmed cell death (apoptosis) and necrosis were detected upon exposure to ferric and ferrous ions, although no lethal effects were observed at environmentally relevant nZVI concentrations. The decreased phagocytic activity further confirmed sublethal adverse effects, even after short-term exposure to ferric and ferrous iron. Detection of sublethal effects, including changes in oxidative stress-related markers such as reactive oxygen species and malondialdehyde production revealed that nZVI had minimal impacts on exposed earthworm cells. In comparison to other works, this study provides more details regarding the effects of the individual iron forms associated with nZVI aging and the cell toxicity effects on the specific earthworms’ immune cells that represent a suitable model for nanomaterial testing.

## 1. Introduction

Nanoscale zero-valent iron (nZVI) and derived nanomaterials are widely applied for nanoremediation and have shown the potential to degrade inorganic and organic pollutants in many laboratory experiments and pilot field applications [1,2]. For remediation, nZVI-based materials in the form of highly concentrated suspensions (up to 10–30 g/L) are usually injected into contaminated soil or groundwater [1]. In addition to interacting with the targeted pollutant, nZVI can also interact with resident soil (micro)organisms [3]. It is noteworthy that due to the mobility of nZVI, highly concentrated suspension disperses in the groundwater aquifer after application and within a relatively short time period the local, environmentally relevant concentrations of this nanomaterial are in the range of mg/L [4,5]. Over the past decade, many authors have studied the negative effects of nZVI and materials derived from it on potentially exposed organisms [6]. The majority of these studies were mainly focused on toxicity and the mechanisms of adverse effects in exposed microorganisms [7]. Only a few authors have explored the effects of nZVI on earthworms and, due to the heterogeneity of nanomaterial types (size, crystallinity, and coating), results have varied greatly [8,9,10,11,12,13,14]. Using OECD tests, some of the authors were able to observe a decrease in the viability, body weight, cocoons production, growth, and reproduction rate of earthworms after exposure to nZVI synthesized by the borohydride method [8,9,11,12]. However, different types of nZVI at similar concentrations did not cause any changes in earthworm viability [13,14]. Intrinsically, nZVI is a reactive material with a high reduction capacity, which can result in reactive oxygen species (ROS) generation and oxidative stress induction [15]. Many authors have already described the oxidative stress and associated cellular damage that occurs in microorganisms after exposure to nZVI [16,17]. It is worth noting that only two studies employed oxidative stress determination in earthworms after exposure to nZVI [11,14]. Earthworm immune cells float freely in the coelomic fluid, where they encounter pollutants, bacteria, viruses and nanoparticles (NPs). These cells are divided into two main populations, eleocytes (free chloragogen cells with mainly nutritive function) and amoebocytes (hyaline and granular immune effector cells) and are frequently used for in vitro toxicity studies of pesticides, heavy metals or nanoparticles [18,19,20,21,22]. For example, Hayashi and Bigorgne described how Ag and TiO_2_ nanoparticles induce oxidative stress and affect the immune function of these cells, demonstrating their potential for studying the fate and adverse effects of new nanomaterials [23,24].

When it is used, nZVI interacts with targeted pollutant molecules and untargeted compounds dissolved in the groundwater or naturally present in the soil [25,26]. This process of oxidation—the transformation of elemental iron to iron oxides and hydroxides—is called aging. Aggregation of nanoparticles is one of the most crucial factors affecting their toxicity, as an increase in their size results in decreased bioavailability and loss of their unique properties at the nano level. Therefore, direct characterization of nanomaterials in the exposure medium is necessary to understand the mechanisms of toxicity and avoid data misinterpretation. It was recently demonstrated that the aging process is accompanied by strong morphological nanomaterial changes, aggregate formation, soluble iron release, and a marked decrease in the toxicity of nZVI to bacterial species [25,27]. The effect of nZVI and its aggregates/aging products on earthworms is still not well described. Specifically, the toxicity of soluble forms of iron released from nZVI particles during the aging process to earthworms is still unknown. For this reason, the present work studies the sublethal effects of nZVI on the two subtypes of coelomocytes with a focus on the role of iron ions in oxidative stress and overall toxicity during the aging process.

## 2. Materials and Methods

### 2.1. Zero-Valent Iron Nanoparticles (nZVI NPs)

In the present study only one type of commercially available, air-stable modified form of nZVI was tested (NANOFER STAR; NANO IRON, Czech Republic). The surface-passivated nZVI particles were synthesized by solid–gas thermal reduction of an iron oxide precursor and partially oxidized to achieve stability in air. The synthesis and characterization of the particles used were recently described in detail by Kaslik et al. (2018) [28]. Briefly, NANOFER STAR particles are core-shell spherical particles that reach a size of approximately 70 nm and are composed of a core of elemental iron (approximately 90%) and a 4-nm-thick shell predominantly made of magnetite (Fe_3_O_4_). 

### 2.2. Characterization of nZVI and Quantification of Soluble Iron Species

The aggregation of nZVI NPs was analyzed by scanning electron microscopy (SEM). The aggregation experiments were performed under the same conditions as the exposure experiments to reflect the state of nanoparticles during the exposure (darkness, 20 °C, 0 and 24 h, distilled water and RPMI 1640 medium; Lonza, Walkersville, MD, USA). After the experiments, the samples of nZVI and RPMI 1640 medium + nZVI NPs were passed through filters to retain the particles. A glass vacuum filtration device (Advantec MFS, Inc., Tokyo, Japan) and a stainless-steel manifold (Speed Flow, Crami Group Srl, Milan, Italy) connected to a vacuum pump (Rocker 300, Rocker Scientific Co., Taiwan, ROC) were used. Separate filters were prepared for quantitative analysis of the particles by SEM. For SEM, polytetrafluoroethylene (PTFE) membrane filters (diameter of 13 mm; Merck Millipore, ME, USA) with a 0.2-μm pore size were used. Before analysis, the filters were dried in an oven (30 °C, 30 min) and stored in capped glass Petri dishes in a desiccator. A Vega high-resolution scanning electron microscope (Tescan, Brno, Czech Republic) was used to determine the number and size of the particles on the filters. Three cut-outs (3 × 8 mm) from each filter were prepared and analyzed. Prior to imaging, a conductive gold layer was sputtered onto the filter cut-outs. Images were then taken with an optimized acceleration voltage of 10 kV and detector working distance of approximately 9 mm. The number and size of the particles from each cut-out were determined by using SigmaScan 5 software (Systat Software, Inc., San Jose, CA, USA), and the results were then recalculated for the whole filter area. The SEM analyses were performed with 100 mg/L nZVI NPs dispersed in distilled water and RPMI 1640 cultivation medium at 2, 6, and 24 h. Fe concentrations were measured using inductively coupled plasma optical emission spectrometry (ICP-OES, 5110 Series, Agilent Technologies, Santa Clara, CA, USA). To separate undissolved NPs, the samples were centrifuged three times at 10,000 g for 10 min. Fe measurements were conducted in triplicate with errors at less than 2%. A calibration standard (Astasol) was purchased from ANALYTIKA, spol. s r. o. (Prague, Czech Republic). The range of calibration of Fe concentrations was 0.02–5 mg/L.

### 2.3. Earthworms and Coelomocytes Extrusion

The *Eisenia andrei* earthworms were collected from our laboratory vermicompost breeding. Adult earthworms with clitella were used for the extrusion of earthworm coelomocytes. Prior to coelomocytes extrusion, the earthworms were kept on wet filter paper for 48 h to clean their gut contents. Then, coelomocytes were extracted via a noninvasive method. Briefly, the earthworms were placed into a falcon tube andextrusion buffer (50.4 mM guaiacol glyceryl ether (GGE; Sigma-Aldrich; Steinheim, Germany) and 5.37 mM EDTA (Sigma-Aldrich, Steinheim, Germany) in PBS 3:2; 2 mL per earthworm) was applied to the earthworms for 2 min. Then, the earthworms were removed from the falcon tube and the collected cells were kept on ice before the following washing steps. The coelomocytes were washed twice with PBS (3:2, 176 mOsm, pH 7.3; 200× *g*, 4 °C, 10 min). RPMI 1640 medium supplemented with 5% heat-inactivated fetal bovine serum (FBS; Life technologies, Carlsbad, CA, USA), 1 M HEPES (Sigma-Aldrich; Gillingham, UK), 100 mM sodium pyruvate (Sigma-Aldrich, Steinheim, Germany), 100 mg/mL gentamycin (Corning, Manassas, VA, USA) and antibiotic-antimycotic solution (Sigma-Aldrich, Steinheim, Germany) was used for cell culture. The culture medium was diluted to 60% (*v*/*v*) with autoclaved milli-Q water (0.05 µS/cm) [29]. Later, nZVI NPs were freshly suspended in water and dispersed in an ultrasound bath (15 min, 300 W, 38 kHz). The nZVI NPs were then vortexed prior to being resuspended in the culture medium (1, 10, and 100 mg/L nZVI NPs). Coelomocytes were incubated in the dark at 20 °C for 2, 6 and 24 h in triplicate (96 well plates, total volume of 100 μL, phagocytosis and apoptosis: 2 × 10^5^ cells/well, ROS quatification: 1 × 10^5^ cells/well). 

### 2.4. Quantification of Reactive Oxygen Species and Lipid Peroxidation

After incubation with 1, 10 and 100 mg/L nZVI NPs for 2, 6 and 24 h, the cells were washed with PBS (3:2; 200× *g*, 4 °C, 10 min); then, 2′,7′-dichlorofluorescein diacetate (DCF-DA; 1:1000 in PBS 3:2; Sigma-Aldrich; Steinheim, Germany) was applied to the cells for 15 min. Afterwards, the cells were washed twice with PBS (3:2; 200× *g*, 4 °C, 10 min). Then, the samples were stained with propidium iodide (PI, 1 mg/L; Sigma-Aldrich; Steinheim, Germany) and analyzed by flow cytometry. To quantify the lipid peroxidation induced by nZVI NPs, a previously optimized protocol for measuring malondialdehyde (MDA) levels in bacterial cultures was used with slight modification to examine earthworm cells [16]. The method is based on quantification of the MDA complex with thiobarbituric acid by HPLC-FLD. Earthworm coelomocytes (1 million cells/mL) were exposed to 3 different concentrations of nZVI NPs (1, 10, and 100 mg/L) and two different ionic forms of iron (FeCl_2_ and Fe_2_(SO_4_)_3_; 100 mg/L). Following exposure, all samples were frozen, stored at −20 °C and analyzed within 2 weeks by high-performance liquid chromatography with fluorescence detection (HPLC-FLD, Waters, Milford, MA, USA). Using external calibration, the final results were determined and are expressed as the production of MDA [nmol/L] per 1 million cells.

### 2.5. Phagocytosis

The phagocytic assay was performed with fluorescent beads (Fluoresbrite^®^ Plain YG; 1 µm microspheres diameter; Polysciencies, Inc., Warrington, PA, UK). After incubation with 1, 10 and 100 mg/L nZVI NPs after 2, 6 and 24 h, the beads were added at a ratio of 1:100 (cells:beads) and incubated for 18 h at 17 °C. After incubation, the cells were washed twice with PBS (3:2; 200× *g*, 4 °C, 10 min), stained with PI (1 mg/L) and analyzed by flow cytometry. Finally, the phagocytosis is expressed as a percentage of engulfed fluorescence beads by the cells.

### 2.6. Viability, Apoptosis, and Necrosis Analyses

Viability and cell death stage assays were performed after incubation of the coelomocytes with 1, 10 and 100 mg/L nZVI NPs. The cell suspension was washed twice with Annexin V buffer (200× *g*, 4 °C, 10 min; 0.01M Hepes (pH 7.4), 0.14M NaCl, and 2.5 mM CaCl_2_ solution) and stained with 30 µL of Annexin V (Thermo Fisher Scientific, Eugene, OR, USA) for 15 min in the dark at room temperature. Prior to flow cytometry analysis, the suspended cells were stained with PI (1 mg/L; Sigma-Aldrich; Steinheim, Germany).

### 2.7. Flow Cytometry

Cellular ROS production, viability, phagocytic activity, and the stages of cell death were analyzed with a flow cytometer (LSR II; BD biosciences, San Jose, CA, USA). To assess the reliability of these assays, 10 mM and 100 mM H_2_O_2_ (Lachner, Neratovice, Czech Republic) were used as positive controls for the endpoints measured by flow cytometry. The forward and side scatter and stain fluorescence settings were adjusted for each coelomocyte subtype and fluorescent probe (Figure S1). For analysis, 10,000 events were detected, and the obtained data were analyzed using FlowJo (9.9.4 version, BD Biosciences, San Jose, CA, USA). Each experiment was performed in triplicate for each component/substance, and the calculated standard deviation is plotted in the figures. For postprocessing the statistical analysis, two-way ANOVA and Bonferroni’s post test were used (* *p* < 0.05, ** *p* < 0.01, *** *p* < 0.001) by GraphPad Prism (8.3.1 version, San Diego, CA, USA)

## 3. Results and Discussion

### 3.1. Particle Aggregation and Iron Release

**Figure 1:**
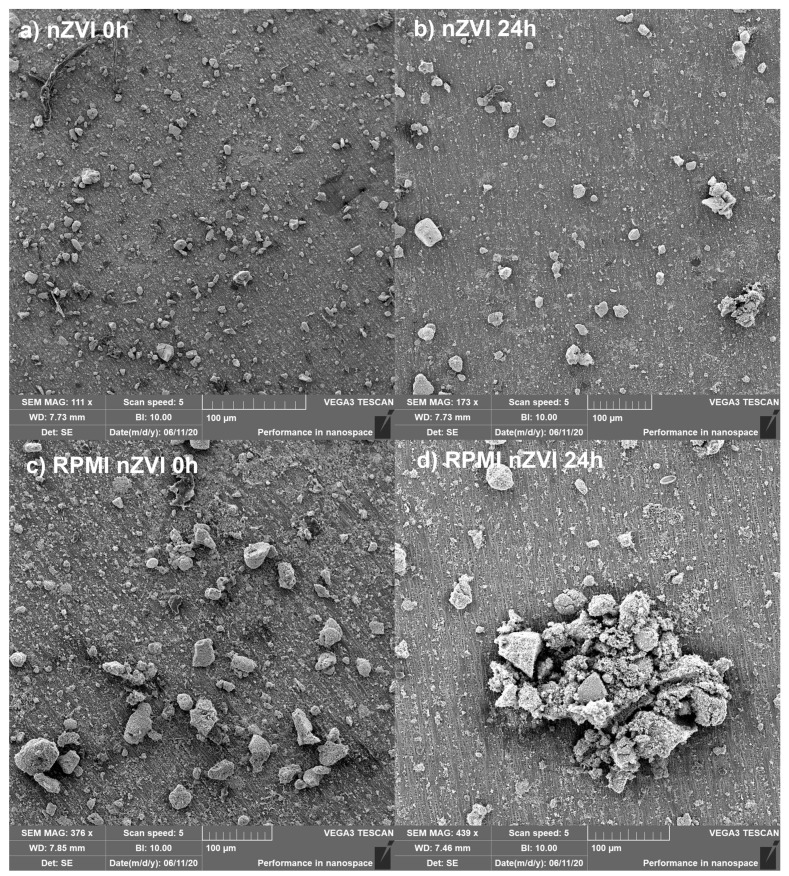
Scanning electron microscopy (SEM) images of fresh and aged nanoscale zero-valent iron (nZVI) in water and in the exposure medium (RPMI 1640): (**a**) Freshly prepared nZVI in distilled water; (**b**) nZVI aged for 24 h in distilled water; (**c**) Freshly prepared nZVI in RPMI 1640 medium; (**d**) nZVI aged for 24 h in RPMI 1640 medium.

**Figure 2:**
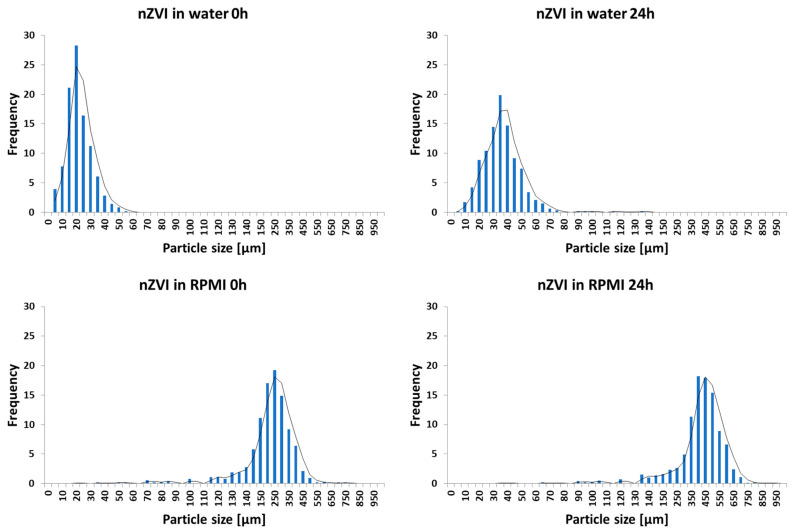
Particle size distribution of fresh and aged nZVI in distilled water and the exposure medium (RPMI 1640).

The results of SEM examination revealed an increase in aggregate formation between fresh nZVI NPs and nZVI NPs aged for 24 h in distilled water (Figure 1a,b). However, the aggregation process was much more intense upon RPMI 1640 medium exposure than in distilled water (Figure 1c,d). Analysis of particle size distribution confirmed an increase in aggregate formation over time and revealed a higher aggregation rate in the RPMI 1640 medium than in distilled water (Figure 2). The aggregate size reached values of 19.68 ± 2.77, 33.83 ± 4.11, 228.44 ± 11.30, and 359.06 ± 16.82 μm for fresh nZVI in distilled water, aged nZVI (24 h) in distilled water, fresh nZVI in RPMI 1640 medium, and aged nZVI (24 h) in RPMI 1640 medium, respectively. nZVI particles aggregated and formed larger clusters after exposure, suggesting that after the application of nZVI NPs in remediation practices, rapid aggregation can be expected depending on the complexity of the soil/groundwater [30,31,32]. On the other hand, the composition of the exposure media, especially the protein content, could also affect the behavior of NPs such as aggregation, bioavailability, and respective toxicity [33]. Many different proteins present in biological tissues/media could form a protein corona around NPs that affects NPs’ behavior and its transport into the cells [34]. The RPMI 1640 medium has been supplemented with FBS which contains mainly bovine serum albumin and a great variety of other proteins. One of the possible explanations of the nZVI aggregation in RPMI 1640 medium was the presence of albumin (and other proteins) as it was documented in a study with TiO_2_ NPs conducted by Márquez et al. (2017) [35].

**Figure 3:**
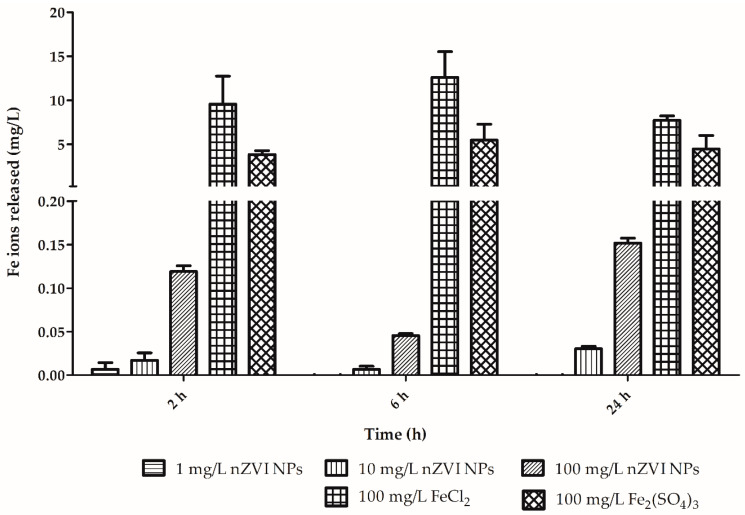
Soluble iron released from different sources during exposure in RPMI 1640 medium.

Moreover, ICP-OES analysis of dissolved iron demonstrated that ions were released from the nanomaterial (Figure 3). The dissolved concentrations corresponded to only approximately 0.1–0.2% of the original amount of nZVI NPs depending on the duration of exposure in RPMI 1640 medium. The analysis of the dissolved iron forms revealed that the concentration in case of nZVI reached maximally 0.15 mg/L in comparison with 7.72 to 9.56 and 3.82 to 4.46 mg/L detected from FeCl_2_ (source of Fe^2+^ ions^)^ and Fe_2_(SO_4_)_3_ (source of Fe^3+^ ions). Notably, under specific conditions, nZVI can be oxidized/transformed into ferric and ferrous species that can be involved in ROS generation through Fenton-like processes [36,37].

### 3.2. Viability, Apoptosis, and Necrosis

**Figure 4:**
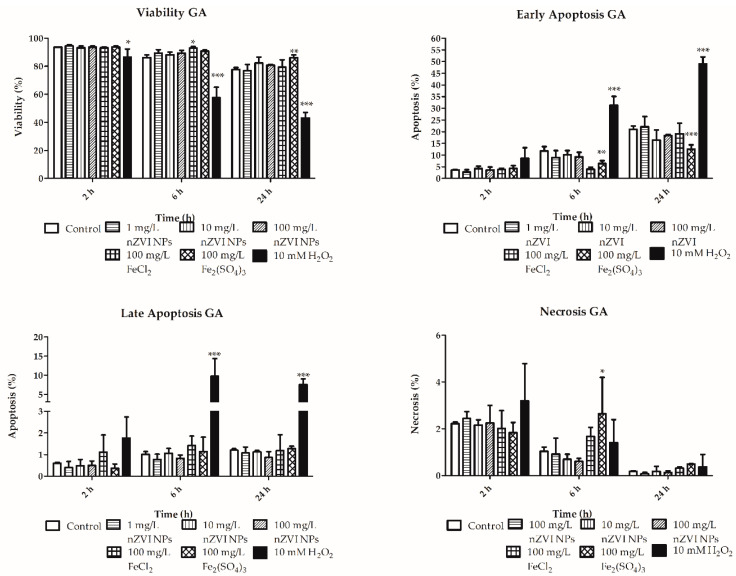
Viability, early and late apoptosis, and necrosis of granular amoebocytes (GA) after exposure to nZVI NPs, FeCl_2_ and Fe_2_(SO_4_)_3_ for 2, 6 and 24 h; 10 mM H_2_O_2_ was used as a positive control; * *p* < 0.05, ** *p* < 0.01, *** *p* < 0.001.

**Figure 5:**
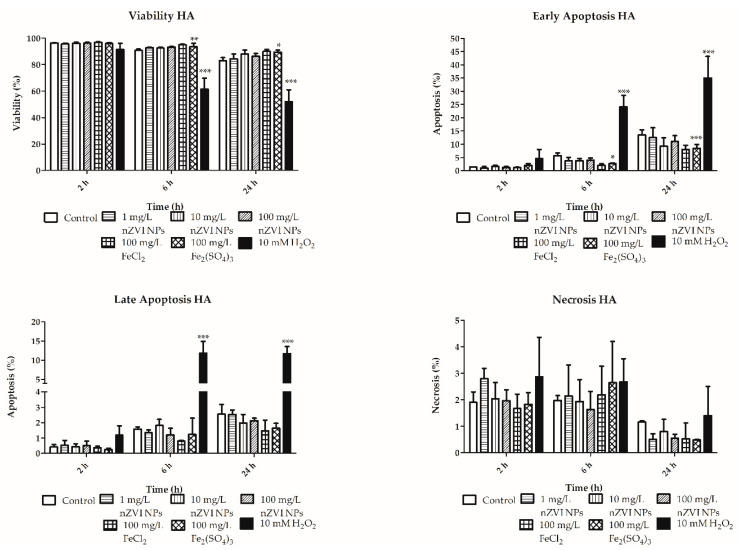
Viability, early and late apoptosis, and necrosis of hyaline amoebocytes (HA) after exposure to nZVI NPs, FeCl_2_ and Fe_2_(SO_4_)_3_ for 2, 6 and 24 h; 10 mM H_2_O_2_ was used as a positive control; * *p* < 0.05, ** *p* < 0.01, *** *p* < 0.001.

Viability analysis did not show any significant effect following exposure of both granular and hyaline amoebocytes to either nZVI NPs at the tested concentrations or the tested ionic forms of iron (Figure 4; Figure 5; Figures S2–S8). The absence of a significant decrease in earthworm cell viability at concentrations of up to 100 mg/L is consistent with previous in vivo studies demonstrating no effect on whole organisms at these levels. Yirsaw et al. (2016) did not observe decreased survival in 3 different soils at concentrations of up to 3 g/kg [14]. However, it is worth noting that other authors have tested different types of nZVI NPs and exposure setups, which resulted in a significant decrease in the viability and body weight of exposed earthworms [8,9,10]. One possible explanation for the contradictory results of these published works is the formation of heterogeneous aggregates and, thus, different cellular (or animal) uptake due to differences in nZVI NPs aggregation rate [30,38]. The wide variety of modified forms of nZVI NPs being tested currently further complicates comparisons of the results; however, based on the results of previous studies, it is clear that nZVI NPs can be toxic to earthworms [8,9,11,12].

In addition to viability studies, the use of flow cytometry allowed the monitoring of cell death stages, including apoptosis and necrosis, in coelomocytes. Moreover, based on the combination of different staining (PI and Annexin V) for the detection of membrane integrity and lipid phosphatidyl serine externalization (a typical marker of apoptosis), the early and late apoptosis were determined in the exposed cells. Only the ferric form of iron significantly decreased early apoptosis of granular amoebocytes (after 6 and 24 h) and increased their necrosis after 6 h (Figure 4). In contrast, no significant effect was observed at any of the tested concentrations of nZVI NPs or ferrous ions. Similar trends in early apoptosis were observed in hyaline amoebocytes, in which ferric iron also induced significant decreases after 6 and 24 h (Figure 5). Despite several changes in the cell death cycle induced by ionic forms of iron, no significant negative effects of nZVI NPs were observed in either type of amoebocytes at environmentally relevant concentrations. The absence of lethal effects is in accordance with in vivo studies focused on the toxicity of nZVI-based material in earthworms and other terrestrial organisms [13,14].

### 3.3. Sublethal Effects: Reactive Oxygen Species, Lipid Peroxidation, and Phagocytosis

**Figure 6:**
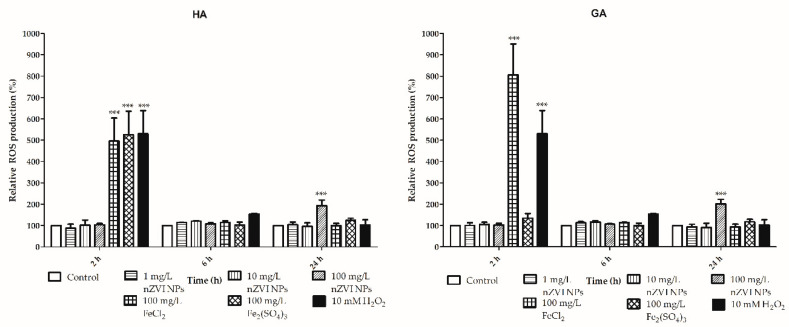
Reactive oxygen species (ROS) generation in granular (GA) and hyaline (HA) amoebocytes after exposure to nZVI NPs, FeCl_2_ and Fe_2_(SO_4_)_3_; 10 mM H_2_O_2_ was used as a positive control; *** *p* < 0.001.

Despite the fact that Yirsaw et al. (2016) did not observe a decrease in the survival of earthworms exposed to nZVI NPs, they were able to detect various concentration-dependent sublethal effects, such as lipid peroxidation and DNA damage [14]. In the current work, we performed a deeper exploration of the sublethal effects of nZVI NPs using the described in vitro approach. ROS level determination in coelomocytes after different treatments revealed an interesting trend that was similar for both types of earthworm immune effector cells (See Figure 6; Figures S8–S13). The highest level of ROS formation was detected in hyaline amoebocytes exposed to Fe^2+^ and Fe^3+^ for 2 h and in granular amoebocytes exposed to Fe^3+^ for the same amount of time. The differences in ROS production between GA and HA caused by Fe^2+^ and Fe^3+^ could be explained by different sensitivities of the two cell types towards various redox forms of iron ions. Different antioxidative defense, uptake and biokinetics could result in different ROS production induced by Fe^2+^ and Fe^3+^. Interestingly, after longer exposure (6 or 24 h), the production of ROS species decreased and did not differ significantly from that in the untreated controls. Several authors have found downward trends in ROS generation following longer exposure periods, and one possible explanation for this phenomenon is the activation of the antioxidative defense system [39,40]. Cellular metabolism involves many mechanisms for the detoxification of ROS that are triggered by oxidative stress. For example, coelomocytes express numerous antioxidative enzymes that protect the cells against the effects of oxidative stress. Metallothioneins are proteins produced by amoebocytes that are induced when metal-oxidative stress occurs [23,41]. The exposure of coelomocytes to nZVI NPs could induce the production of metallothioneins to protect the cells against the oxidative stress exerted by iron ions or a highly reactive nanomaterial. Moreover, there are other antioxidant enzymes (catalase and superoxide dismutase), that are activated in response to an imbalance in ROS metabolism. However, ROS formation in hyaline and granular amoebocytes after exposure to nZVI NPs showed a different trend. As observed with the DCF-DA test which measures the intracellular ROS, nZVI at 100 mg/L significantly increased nonspecific ROS generation after the longer exposure period of 24 h in both amoebocyte subpopulations. This opposite trend in ROS formation in cells after exposure to nZVI NPs could be explained by delayed cellular uptake of nanoparticles compared to iron ions or by dissolution/degradation of the oxidic shell [42]. These results emphasize the need for a further investigation of longer exposure times using an approach other than primary culture, which has a limited viability of approximately 24 h.

**Figure 7:**
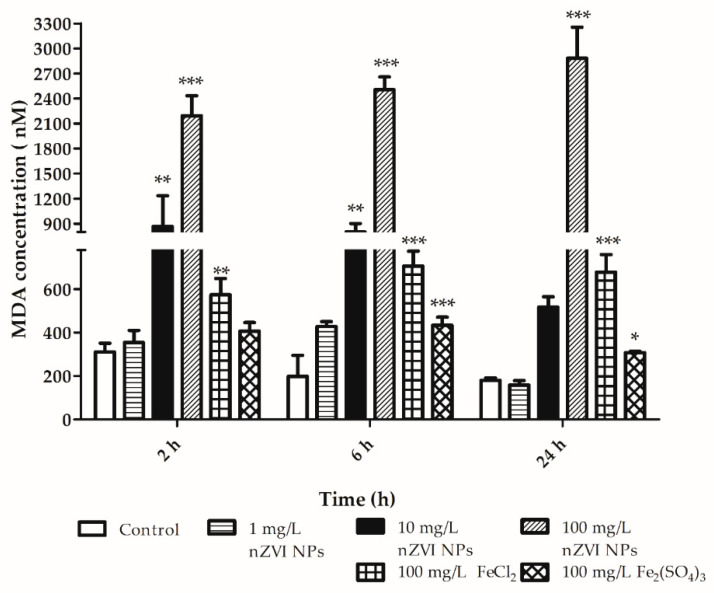
Malondialdehyde (MDA) formation in amoebocytes after exposure to nZVI NPs, FeCl_2_ and Fe_2_(SO_4_)_3_ for 2, 6 and 24 h; * *p* < 0.05, ** *p* < 0.01, *** *p* < 0.001.

Analysis of lipid peroxidation in coelomocytes after exposure to nZVI NPs showed a dose-dependent increase in MDA production (Figure 7). Even the tested concentration 10 mg/L of nZVI NPs induced a significant elevation of MDA levels compared to that in the control sample without the nanomaterial. Moreover, different forms of iron ions (Fe^2+^ and Fe^3+^) also induced a significant increase in lipid peroxidation compared to that in the control sample. The same concentration of iron in the form of ferrous ions (Fe^2+^ dissolved from FeCl_2_) produced significantly more MDA than iron in the form of ferric ions (Fe^3+^ dissolved from Fe_2_(SO_4_)_3_). Determination of MDA levels was the most sensitive assay out of those used, as in a previous study focused on adverse effects in bacteria [27]. The applicability and sensitivity of the MDA determination assay has already been established using earthworms exposed to ZnO NPs, as well as to nZVI NPs [11,43]. The results of the MDA formation assay in amoebocytes exposed to nZVI NPs in the current study are consistent with an in vivo study by Liang et al. (2017) and show the potential of in vitro tests for NPs’ toxicity testing [11].

**Figure 8:**
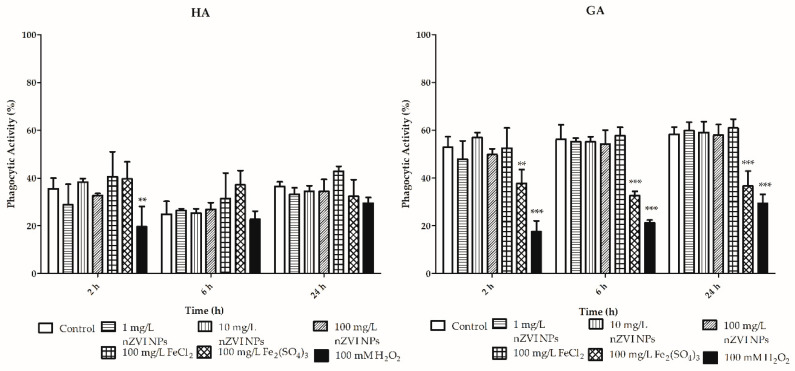
Phagocytic activity of hyaline (HA) and granular (GA) amoebocytes after exposure to nZVI NPs, FeCl_2_ and Fe_2_(SO_4_)_3_ for 2, 6 and 24 h; 100 mM H_2_O_2_ was used as a positive control; ** *p* < 0.01, *** *p* < 0.001.

The phagocytic activity of hyaline amoebocytes was not affected by the tested concentrations of nZVI NPs or iron ions (Figure 8; Figures S14–S19). The granular amoebocytes were more sensitive than the other subtype of coelomocytes. The tested concentrations of ferric iron significantly decreased the phagocytic activity of the exposed granular amoebocytes after 2, 6 and 24 h of exposure. The normal phagocytic activity of the untreated granular amoebocyte subpopulation was 52–58%, while that of granular amoebocytes treated with ferric iron was 37, 32, and 36% at 2, 6, and 24 h, respectively. 

### 3.4. The Effect of Dissolved Iron Species on Toxicity

As previously mentioned, many authors have studied the toxicity of nZVI NPs and elucidated the mechanisms of its adverse effects using microbial species. Despite the many different and contradictory results, the majority of those studies concluded that oxidative stress is one of the main adverse effects induced by nZVI NPs and the materials derived from it [3]. The imbalance in ROS metabolism induced by increased ROS production or ineffective antioxidative defense mechanisms can result in oxidation of cellular biomolecules and subsequent cell death. Many reaction cascades have already been described in which nZVI NPs undergo oxidation/reduction with consequent ROS production [15]. Both ferrous and ferric ions are known to be able to induce intracellular ROS formation via Fenton reactions [44]. The redox reactions of ferrous and ferric ions with hydrogen peroxide result in the formation of hydroxyl and hydroperoxyl radicals, which can then degrade biomolecules such as nucleic acids, proteins, and lipids. Some recent studies performed on bacterial species and human cell lines have suggested that not only highly reactive nZVI NPs but also iron ions dissolved from nanomaterials could play a critical role in mediating the toxicity of nZVI NPs [45,46]. On the other hand, another study showed no significant relationship between toxicity/lipid peroxidation in bacteria and the concentration of dissolved iron from different nZVI-based materials during 2 months of aging [27]. Similarly, another study ascribed greater toxicity to *Escherichia coli* exposed to nZVI NPs than to *Escherichia coli* exposed to the same concentration of ferrous iron under both air-saturated and deaerated conditions [47]. The findings regarding early apoptosis and necrosis of granular amoebocytes exposed to ferric iron in this study are highly consistent with the decrease in phagocytic activity and demonstrate the potential negative effects of dissolved iron in this form (Figure 4 and Figure 5). However, dissolved iron ions reached levels 0.1–0.2% of the amount reached with nZVI NPs in this experiment, which is negligible in comparison to dissolved iron species from FeCl_2_ and Fe_2_(SO_4_)_3_ that reached from 50 to 100 times higher concentrations (see Section 3.1 and Figure 3). It is noteworthy that approximately the same concentration levels of dissolved iron were detected in a previous 2-month aging experiment [27]. Nevertheless, even at such a high nZVI NPs concentration of 10 g/L, which is, from a practical perspective, irrelevant, as it is 100 times higher than the concentration used in our experiment or that is detected at sites of nZVI NPs applications, similar concentrations of ferric and ferrous ions could not theoretically be reached (considering that 0.1–0.2% of iron that is dissolved). This finding indicates that dissolved iron species do not play an important role in toxicity to earthworms in the context of nZVI NPs applications. It is worth noting that under different conditions in heterogenic matrices (e.g., real groundwater, different types of soil, etc.), the aging process is different, and dissolution occurs with different kinetics. Moreover, Fe^0^ could be transformed by microbial species into various insoluble iron species or their oxidation products [25]. Despite the many aforementioned factors affecting the aging processes of nZVI NPs, the results of the current study show that nZVI NPs applications pose minimal risk to earthworms.

## 4. Conclusions

The results of the current study demonstrate the necessity of material characterization in the medium used for exposure conditions to achieve reliable results. While the fact that nZVI NPs aggregated in the RPMI 1640 medium makes the experimental setup inapplicable for determining the toxicity of fresh nZVI NPs, the formation of aggregates reflects the fate of nanoparticles after real application. Evaluation of the toxicity of nZVI NPs in the form of aggregates is equally or even more important than evaluation of freshly prepared particles, which are present in the environment only for several hours or days. Understanding the mechanisms of iron dissolution and ion release, as well as their impact on overall toxicity, can help in the assessment of the environmental safety of nZVI NPs. The toxicological data obtained in this study suggest that two sizes of nZVI NPs aggregates (after 0 and 24 h in RPMI 1640) and ferrous and ferric ions, as potentially released forms of iron, do not significantly affect the viability of coelomocytes and probably do not pose a risk to exposed earthworms. Determination of the sublethal effects including impacts on phagocytosis and oxidative stress-related markers showed that ferric and ferrous ions as well as nZVI NPs aggregates had a slight effect. The combination of the quantification of dissolved iron and the most sensitive assay (from the battery of assay used), malondialdehyde determination, showed that, even in the form of aggregates, nZVI NPs cause higher lipid peroxidation than their potential transformation products (i.e., dissolved ferric and ferrous ions) in earthworm coelomocytes. Overall, the results of the present study confirm the environmental safety of nZVI NPs and suggest that the toxicity of nZVI NPs is potentially reduced during aging. Further studies incorporating the nZVI NPs characterization during the aging process and iron dissolution under different environmental conditions are needed to better understand material behavior and toxicity in real-world nanoremediation applications. Our results also show that the methodological concept of testing isolated immune earthworm cells is a suitable approach to testing for nanomaterials toxicity and to elucidate mechanistical aspects of the nanomaterials with respect to possible different modes of actions.

## Supplementary Materials

The following are available online at https://www.mdpi.com/2079-4991/10/11/2189/s1. Figure S1. Detection of coelomocytes subpopulations through flow cytometry. Coelomocytes populations were detected and divided into eleocytes (E), hyaline (HA) and granular amoebocytes (GA). A) coelomocytes non-treated (control) and coelomocytes exposed to 100 mg/L of nZVI, FeCl_2_ and Fe_2_(SO_4_)_3_; B) RPM 1640 medium and RPMI 1640 medium with 100 mg/L of nZVI, FeCl_2_ and Fe_2_(SO_4_)_3_ without coelomocytes after 6 h. Figure S2. Apoptosis activity of hyaline amoebocytes (HA) without annexin V, without treatment (control) and HA exposed to 10 mM H_2_O_2_ (positive control), 100 mg/L of nZVI, FeCl_2_ and Fe_2_(SO_4_)_3_ after 2 h. Figure S3. Apoptosis activity of granular amoebocytes (GA) without annexin V, without treatment (control) and GA exposed to 10 mM H_2_O_2_ (positive control), 100 mg/L of nZVI, FeCl_2_ and Fe_2_(SO_4_)_3_ after 2 h. Figure S4. Apoptosis activity of hyaline amoebocytes (HA) without annexin V, without treatment (control) and HA exposed to 10 mM H_2_O_2_ (positive control), 100 mg/L of nZVI, FeCl_2_ and Fe_2_(SO_4_)_3_ after 6 h. Figure S5. Apoptosis activity of granular amoebocytes (GA) without annexin V, without treatment (control) and GA exposed to 10 mM H_2_O_2_ (positive control), 100 mg/L of nZVI, FeCl_2_ and Fe_2_(SO_4_)_3_ after 6 h. Figure S6. Apoptosis activity of hyaline amoebocytes (HA) without annexin V, without treatment (control) and HA exposed to 10 mM H_2_O_2_ (positive control), 100 mg/L of nZVI, FeCl_2_ and Fe_2_(SO_4_)_3_ after 24 h. Figure S7. Apoptosis activity of granular amoebocytes (GA) without annexin V, without treatment (control) and GA exposed to 10 mM H_2_O_2_ (positive control), 100 mg/L of nZVI, FeCl_2_ and Fe_2_(SO_4_)_3_ after 24 h. Figure S8. Reactive Oxygen Species production of hyaline amoebocytes (HA) without fluorescence, without treatment (control) and HA exposed to 10 mM H_2_O_2_ (positive control), 100 mg/L of nZVI, FeCl_2_ and Fe_2_(SO_4_)_3_ after 2 h. Figure S9. Reactive Oxygen Species production of granular amoebocytes (GA) without fluorescence, without treatment (control) and GA exposed to 10 mM H_2_O_2_ (positive control), 100 mg/L of nZVI, FeCl_2_ and Fe_2_(SO_4_)_3_ after 2 h. Figure S10. Reactive Oxygen Species production of hyaline amoebocytes (HA) without fluorescence, without treatment (control) and HA exposed to 10 mM H_2_O_2_ (positive control), 100 mg/L of nZVI, FeCl_2_ and Fe_2_(SO_4_)_3_ after 6 h. Figure S11. Reactive Oxygen Species production of granular amoebocytes (GA) without fluorescence, without treatment (control) and GA exposed to 10 mM H_2_O_2_ (positive control), 100 mg/L of nZVI, FeCl_2_ and Fe_2_(SO_4_)_3_ after 6 h. Figure S12. Reactive Oxygen Species production of hyaline amoebocytes (HA) without fluorescence, without treatment (control) and HA exposed to 10 mM H_2_O_2_ (positive control), 100 mg/L of nZVI, FeCl_2_ and Fe_2_(SO_4_)_3_ after 24 h. Figure S13. Reactive Oxygen Species production of granular amoebocytes (GA) without fluorescence, without treatment (control) and GA exposed to 10 mM H_2_O_2_ (positive control), 100 mg/L of nZVI, FeCl_2_ and Fe_2_(SO_4_)_3_ after 24 h. Figure S14. Phagocytic activity of hyaline amoebocytes (HA) without Fluoresbrite, without treatment (control) and HA exposed to 100 mM H_2_O_2_ (positive control), 100 mg/L of nZVI, FeCl_2_ and Fe_2_(SO_4_)_3_ after 2 h. Figure S15. Phagocytic activity of granular amoebocytes (GA) without annexin V, without treatment (control) and GA exposed to 100 mM H_2_O_2_ (positive control), 100 mg/L of nZVI, FeCl_2_ and Fe_2_(SO_4_)_3_ after 2 h. Figure S16. Phagocytic activity of hyaline amoebocytes (HA) without Fluoresbrite, without treatment (control) and HA exposed to 100 mM H_2_O_2_ (positive control), 100 mg/L of nZVI, FeCl_2_ and Fe_2_(SO_4_)_3_ after 6 h. Figure S17. Phagocytic activity of granular amoebocytes (GA) without Fluoresbrite, without treatment (control) and HA exposed to 100 mM H_2_O_2_ (positive control), 100 mg/L of nZVI, FeCl_2_ and Fe_2_(SO_4_)_3_ after 6 h. Figure S18. Phagocytic activity of hyaline amoebocytes (HA) without Fluoresbrite, without treatment (control) and HA exposed to 100 mM H_2_O_2_ (positive control), 100 mg/L of nZVI, FeCl_2_ and Fe_2_(SO_4_)_3_ after 24 h. Figure S19. Phagocytic activity of granular amoebocytes (GA) without Fluoresbrite, without treatment (control) and HA exposed to 100 mM H_2_O_2_ (positive control), 100 mg/L of nZVI, FeCl_2_ and Fe_2_(SO_4_)_3_ after 24 h.
