# Supplementary material for: In Vitro Study of the Toxicity Mechanisms of Nanoscale Zero-Valent Iron (nZVI) and Released Iron Ions Using Earthworm Cells
Source: Nanomaterials (Basel). 2020 Nov 3;10(11):2189. doi: 10.3390/nano10112189 (PMC7692149; doi:10.3390/nano10112189)
Supplement: Supplementary file 1 [file nanomaterials-10-02189-s001.pdf]

nanomaterials

Supplementary Materials

In Vitro Study of the Toxicity Mechanisms of Nanoscale Zero-Valent Iron (nZVI) and Released Iron Ions Using Earthworm Cells

Jaroslav Semerad[1,2], Natividad Isabel Navarro Pacheco[1,3], Alena Grasserova[1,2], Petra Prochazkova[1], Martin Pivokonsky[4], Lenka Pivokonska[4] and Tomas Cajthaml[1,2,*]

[1] Institute of Microbiology of the Czech Academy of Sciences, Vídeňská 1083, CZ-142 20, Prague 4, Czech Republic
[2] Institute for Environmental Studies, Faculty of Science, Charles University, Benátská 2, CZ-128 01, Prague 2, Czech Republic
[3] First Faculty of Medicine, Charles University, Kateřinská 1660/32, CZ-121 08, Prague 2, Czech Republic
[4] Institute of Hydrodynamics of the Czech Academy of Sciences, Pod Paťankou 30/5, CZ-166 12, Prague 6, Czech Republic
* Correspondence: cajthaml@biomed.cas.cz

Electronic Supplementary Information

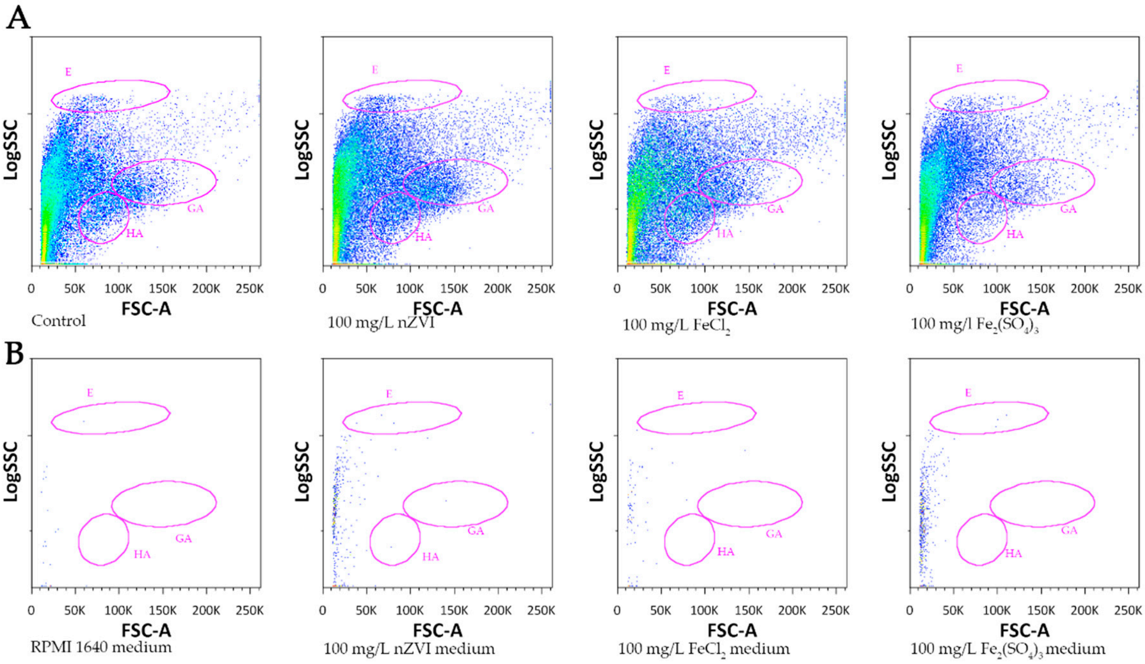

Figure S1. Detection of coelomocytes subpopulations through flow cytometry. Coelomocytes populations were detected and divided into eleocytes (E), hyaline (HA) and granular amoebocytes (GA). (**A**) coelomocytes non-treated (control) and coelomocytes exposed to 100 mg/L of nZVI, $FeCl_2$ and $Fe_2(SO_4)_3$; (B) RPM1640 medium and medium with 100 mg/L of nZVI, $FeCl_2$ and $Fe_2(SO_4)_3$ without coelomocytes after 6 hours.

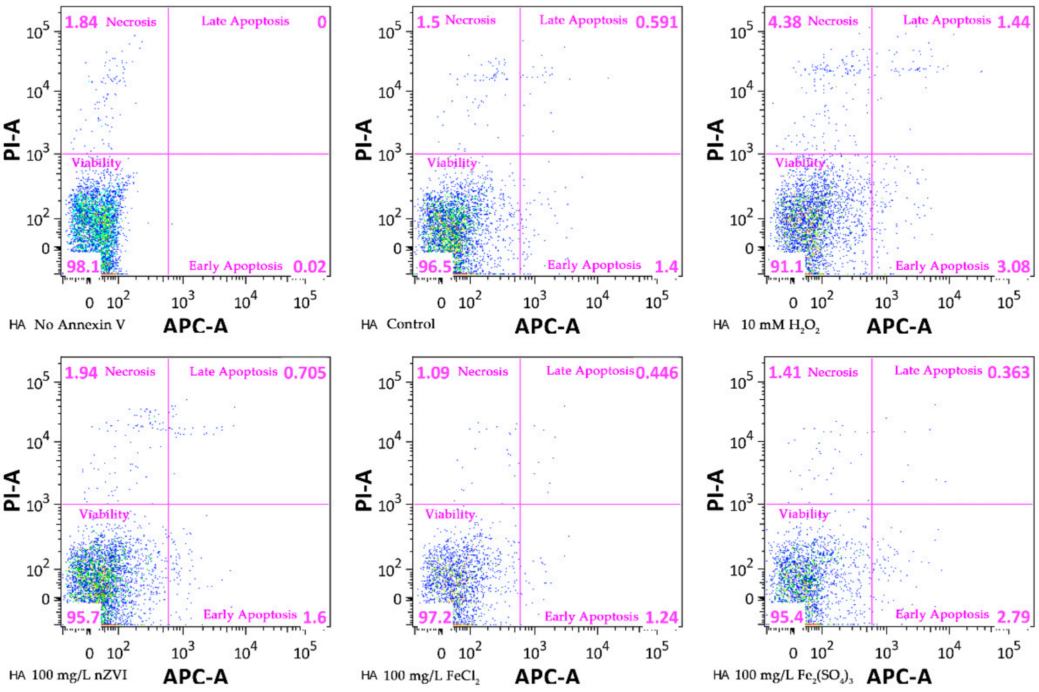

Figure S2. Apoptosis activity of hyaline amoebocytes (HA) without annexin V, without treatment (control) and HA exposed to 10 mM H_2O_2 (positive control), 100 mg/L of nZVI, $FeCl_2$ and $Fe_2(SO_4)_3$ after 2 hours.

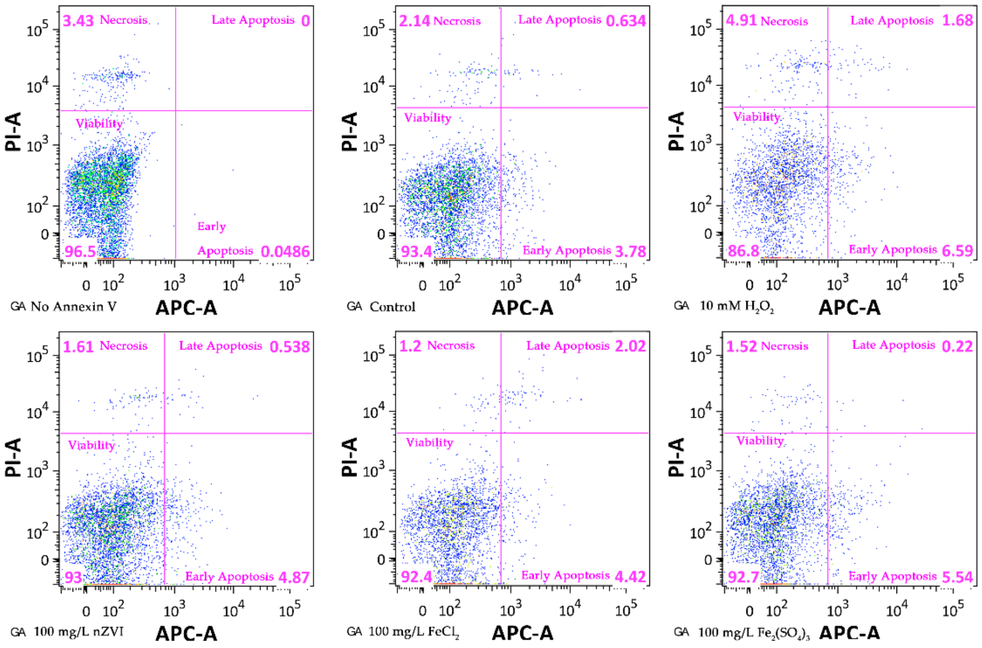

Figure S3. Apoptosis activity of granular amoebocytes (GA) without annexin V, without treatment (control) and GA exposed to 10 mM H_2O_2 (positive control), 100 mg/L of nZVI, $FeCl_2$ and $Fe_2(SO_4)_3$ after 2 hours.

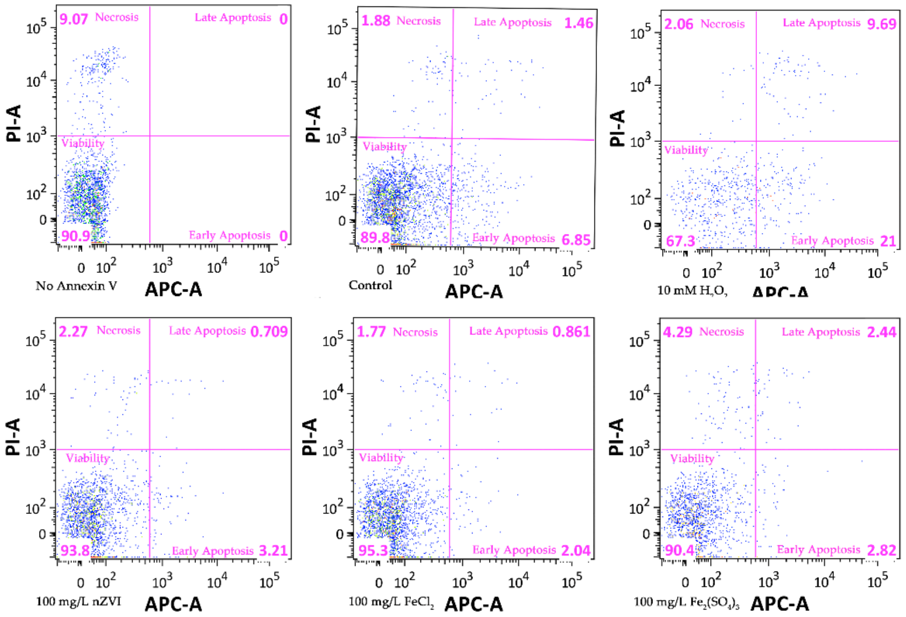

Figure S4. Apoptosis activity of hyaline amoebocytes (HA) without annexin V, without treatment (control) and HA exposed to 10 mM H_2O_2 (positive control), 100 mg/L of nZVI, $FeCl_2$ and $Fe_2(SO_4)_3$ after 6 hours.

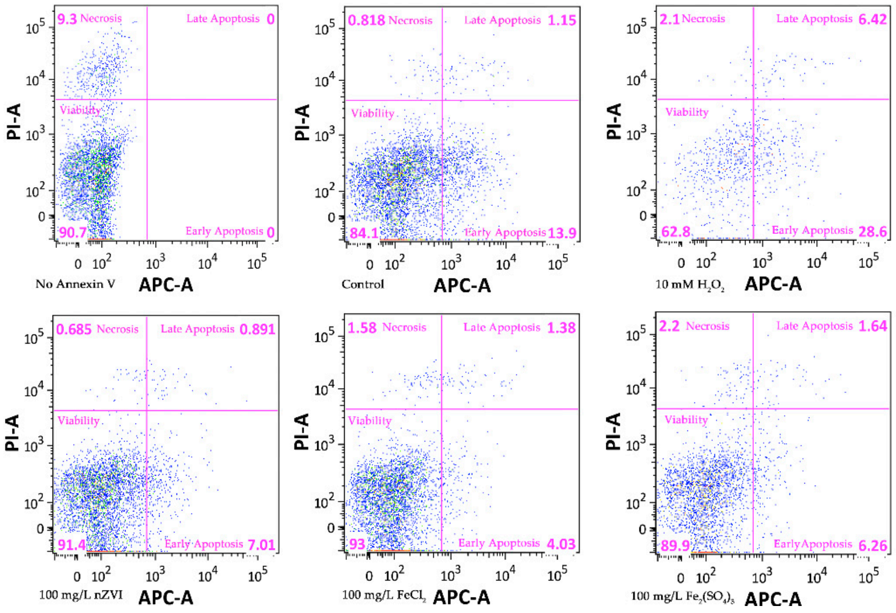

Figure S5. Apoptosis activity of granular amoebocytes (GA) without annexin V, without treatment (control) and GA exposed to 10 mM H_2O_2 (positive control), 100 mg/L of nZVI, $FeCl_2$ and $Fe_2(SO_4)_3$ after 6 hours.

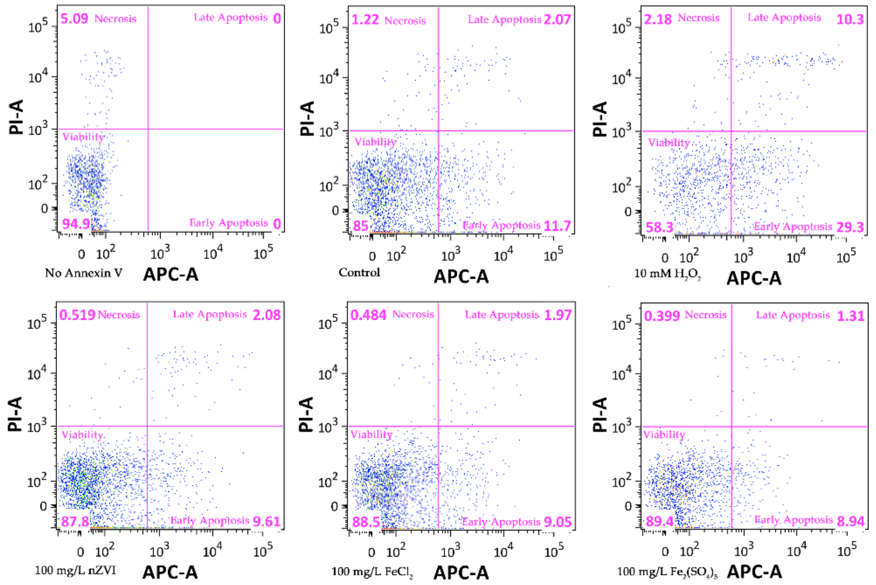

Figure S6. Apoptosis activity of hyaline amoebocytes (HA) without annexin V, without treatment (control) and HA exposed to 10 mM H_2O_2 (positive control), 100 mg/L of nZVI, $FeCl_2$ and $Fe_2(SO_4)_3$ after 24 hours.

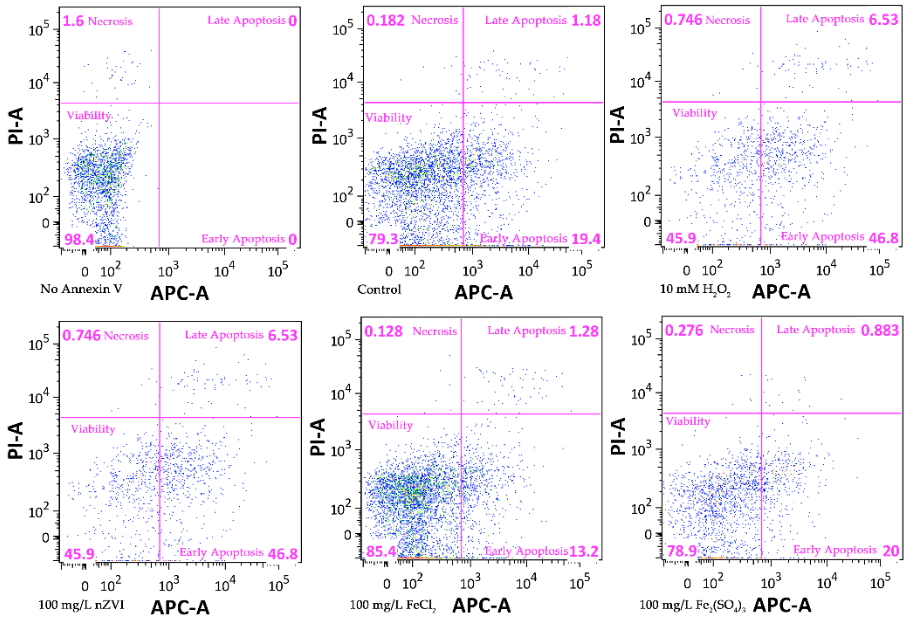

Figure S7. Apoptosis activity of granular amoebocytes (GA) without annexin V, without treatment (control) and GA exposed to 10 mM H_2O_2 (positive control), 100 mg/L of nZVI, $FeCl_2$ and $Fe_2(SO_4)_3$ after 24 hours.

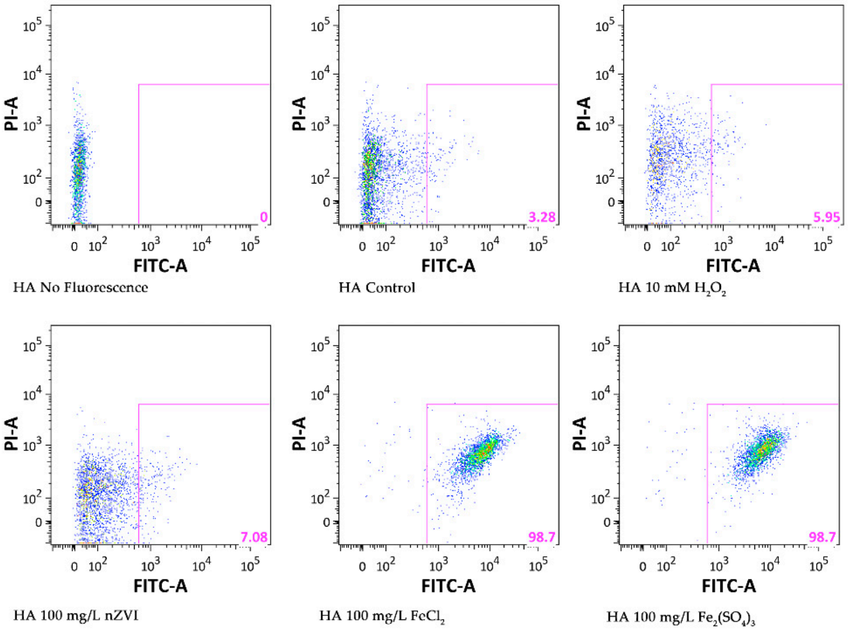

Figure S8. Reactive Oxygen Species production of hyaline amoebocytes (HA) without fluorescence, without treatment (control) and HA exposed to 10 mM H_2O_2 (positive control), 100 mg/L of nZVI, $FeCl_2$ and $Fe_2(SO_4)_3$ after 2 hours.

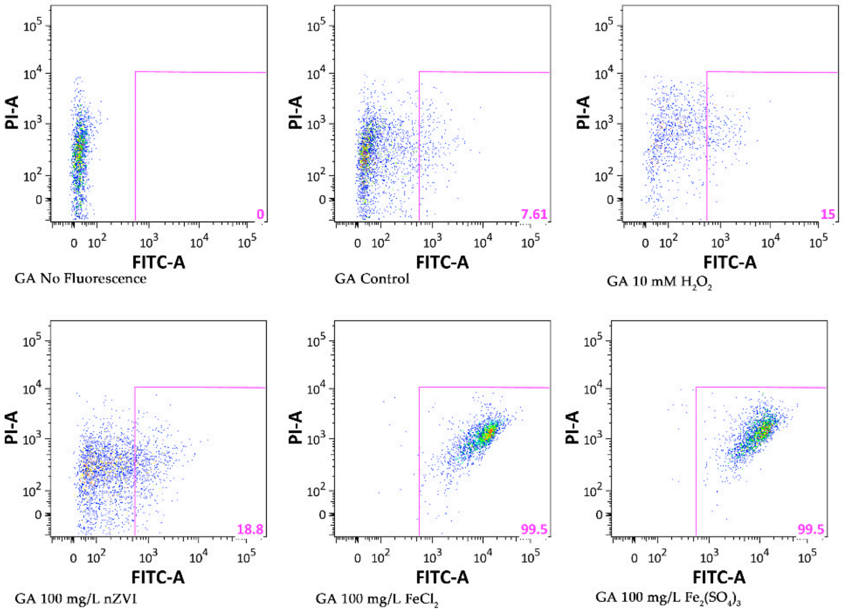

Figure S9. Reactive Oxygen Species production of granular amoebocytes (GA) without fluorescence, without treatment (control) and GA exposed to 10 mM H_2O_2 (positive control), 100 mg/L of nZVI, $FeCl_2$ and $Fe_2(SO_4)_3$ after 2 hours.

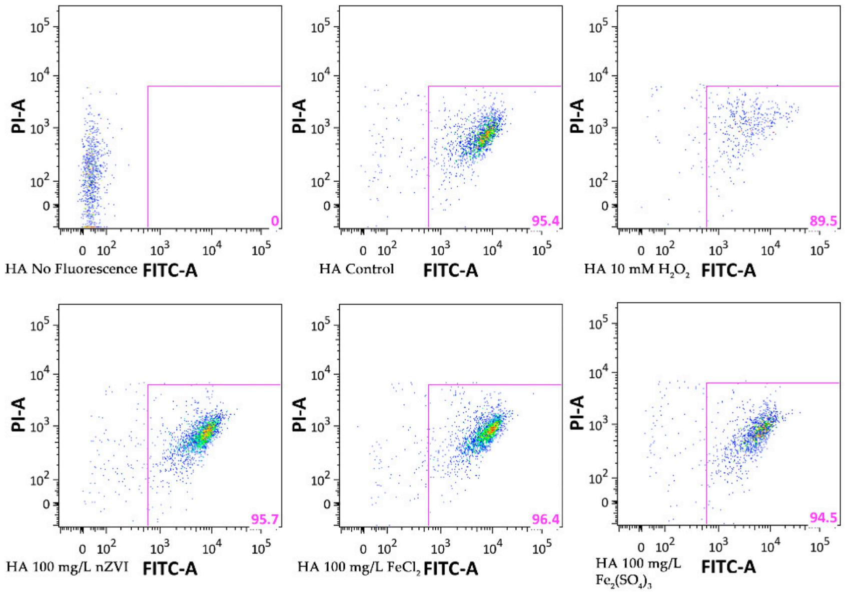

Figure S10. Reactive Oxygen Species production of hyaline amoebocytes (HA) without fluorescence, without treatment (control) and HA exposed to 10 mM H_2O_2 (positive control), 100 mg/L of nZVI, $FeCl_2$ and $Fe_2(SO_4)_3$ after 6 hours.

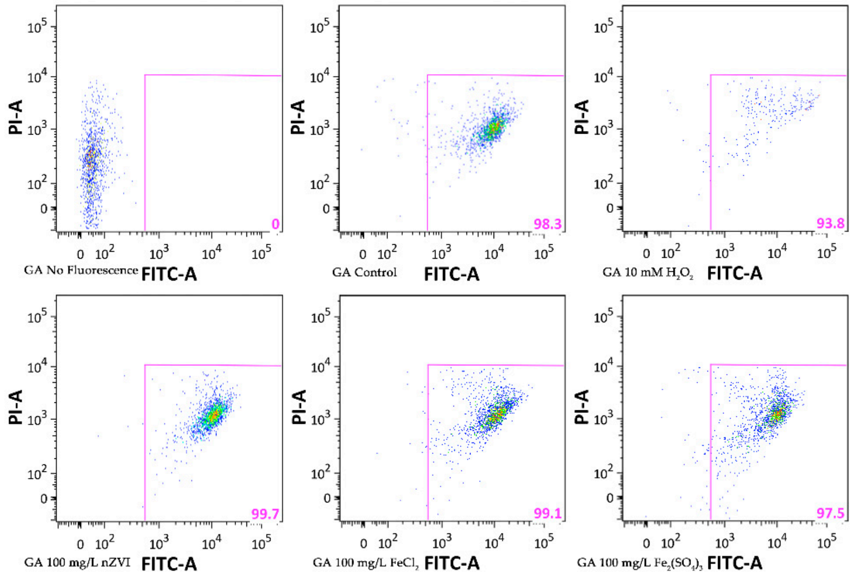

Figure S11. Reactive Oxygen Species production of granular amoebocytes (GA) without fluorescence, without treatment (control) and GA exposed to 10 mM H_2O_2 (positive control), 100 mg/L of nZVI, $FeCl_2$ and $Fe_2(SO_4)_3$ after 6 hours.

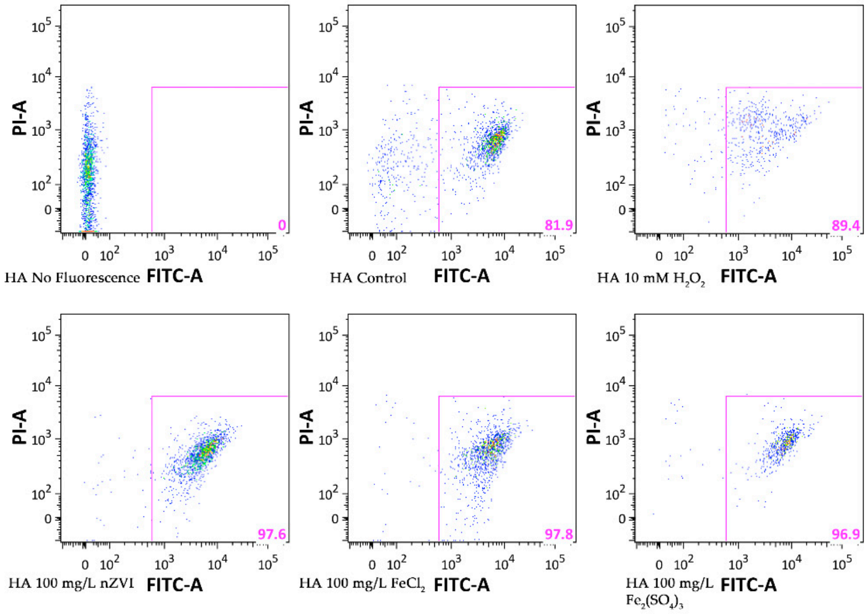

Figure S12. Reactive Oxygen Species production of hyaline amoebocytes (HA) without fluorescence, without treatment (control) and HA exposed to 10 mM H_2O_2 (positive control), 100 mg/L of nZVI, $FeCl_2$ and $Fe_2(SO_4)_3$ after 24 hours.

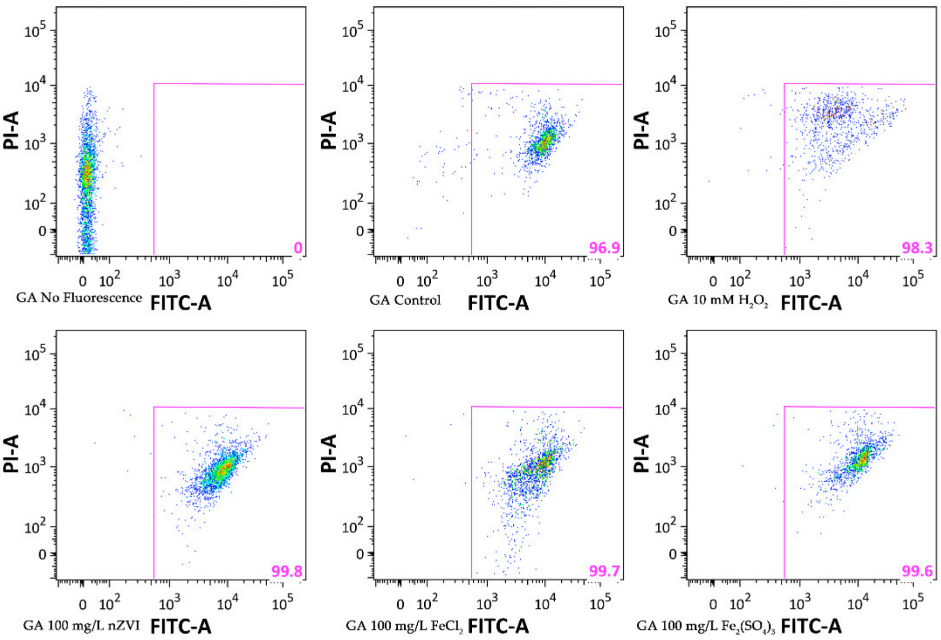

Figure S13. Reactive Oxygen Species production of granular amoebocytes (GA) without fluorescence, without treatment (control) and GA exposed to 10 mM H_2O_2 (positive control), 100 mg/L of nZVI, $FeCl_2$ and $Fe_2(SO_4)_3$ after 24 hours.

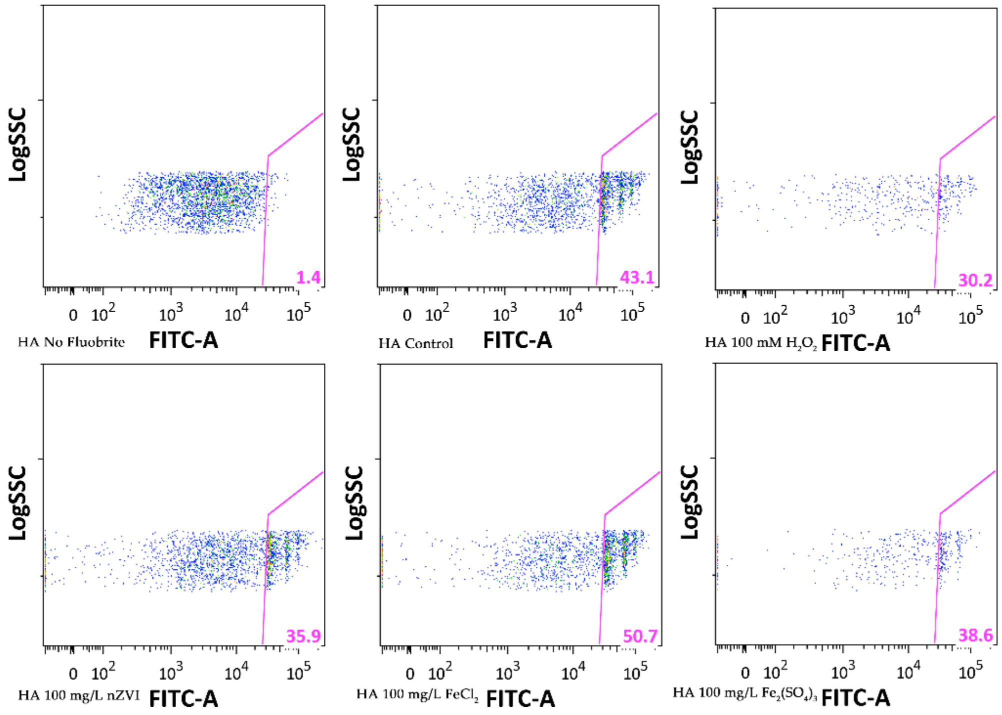

Figure S14. Phagocytic activity of hyaline amoebocytes (HA) without Fluoresbrite, without treatment (control) and HA exposed to 100 mM H_2O_2 (positive control), 100 mg/L of nZVI, $FeCl_2$ and $Fe_2(SO_4)_3$ after 2 hours.

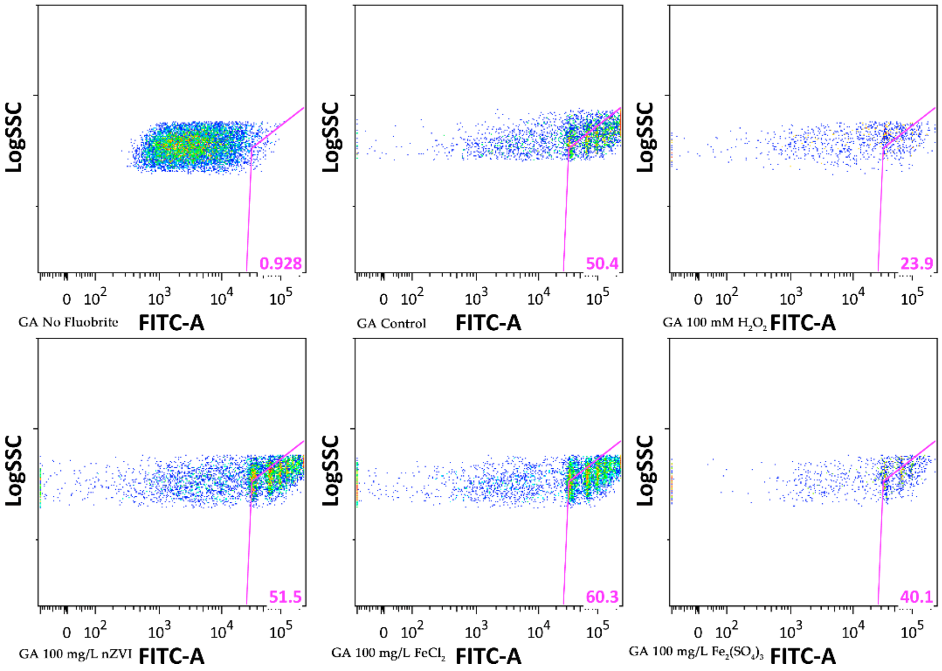

Figure S15. Phagocytic activity of granular amoebocytes (GA) without Fluoresbrite, without treatment (control) and GA exposed to 100 mM H_2O_2 (positive control), 100 mg/L of nZVI, $FeCl_2$ and $Fe_2(SO_4)_3$ after 2 hours.

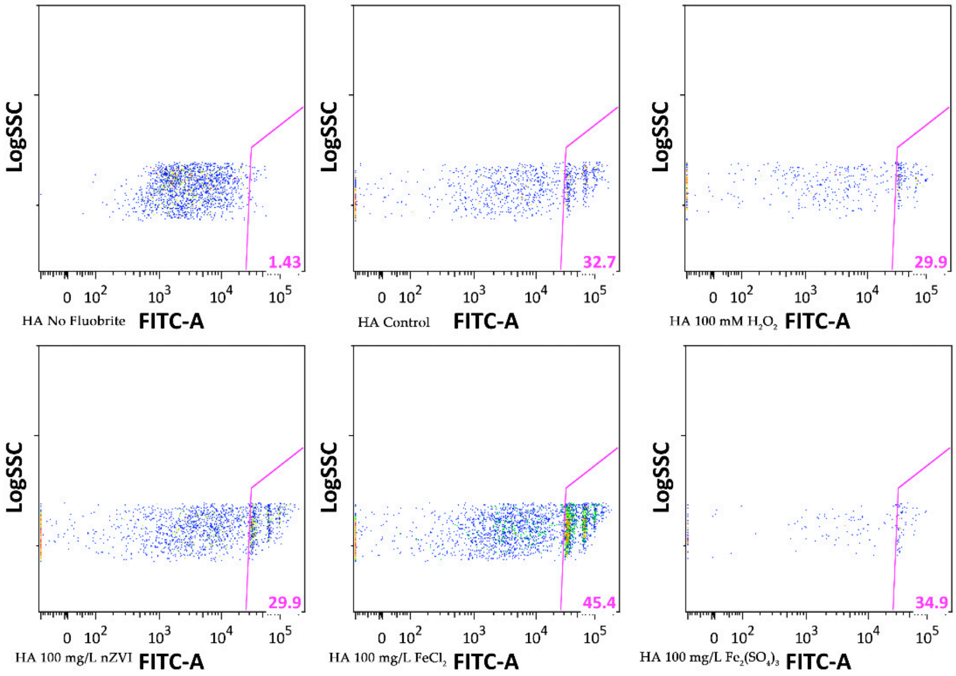

Figure S16. Phagocytic activity of hyaline amoebocytes (HA) without Fluoresbrite, without treatment (control) and HA exposed to 100 mM H_2O_2 (positive control), 100 mg/L of nZVI, $FeCl_2$ and $Fe_2(SO_4)_3$ after 6 hours.

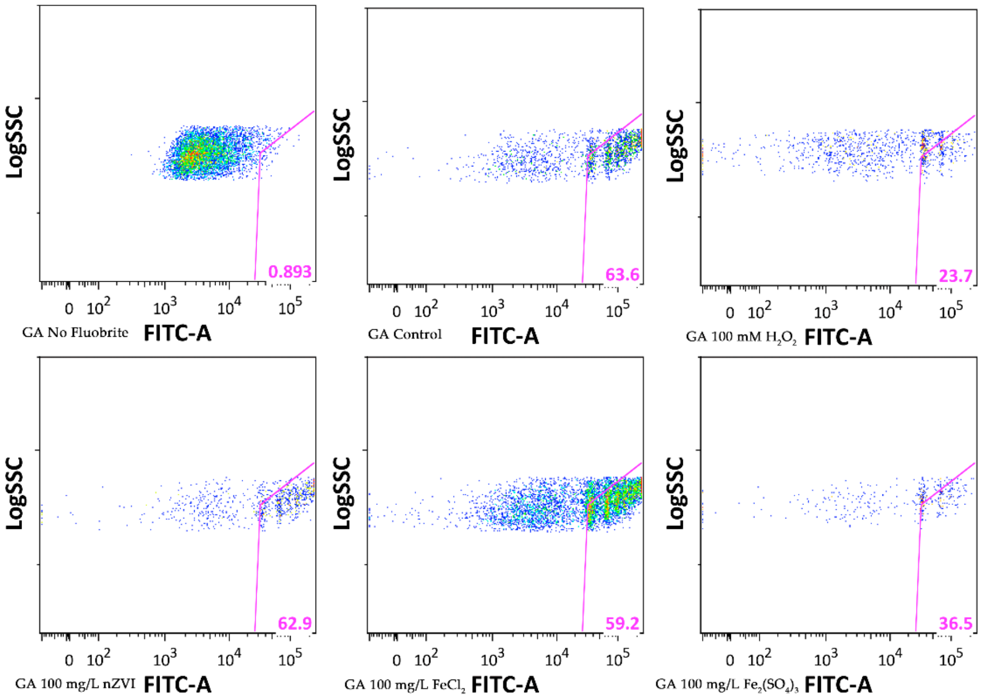

Figure S17. Phagocytic activity of granular amoebocytes (GA) without Fluoresbrite, without treatment (control) and GA exposed to 100 mM H_2O_2 (positive control), 100 mg/L of nZVI, $FeCl_2$ and $Fe_2(SO_4)_3$ after 6 hours.

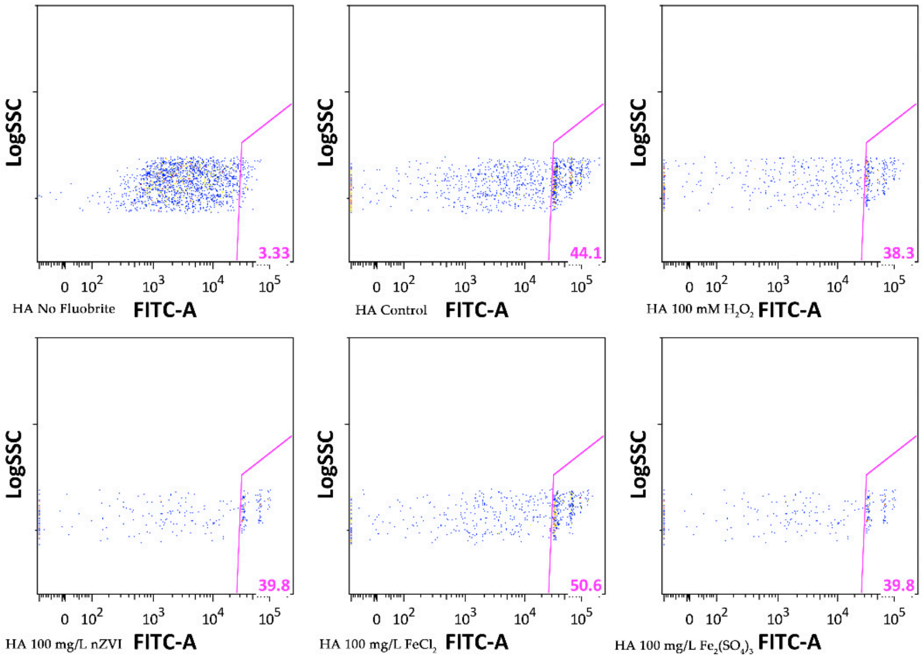

Figure S18. Phagocytic activity of hyaline amoebocytes (HA) without Fluoresbrite, without treatment (control) and HA exposed to 100 mM H_2O_2 (positive control), 100 mg/L of nZVI, $FeCl_2$ and $Fe_2(SO_4)_3$ after 24 hours.

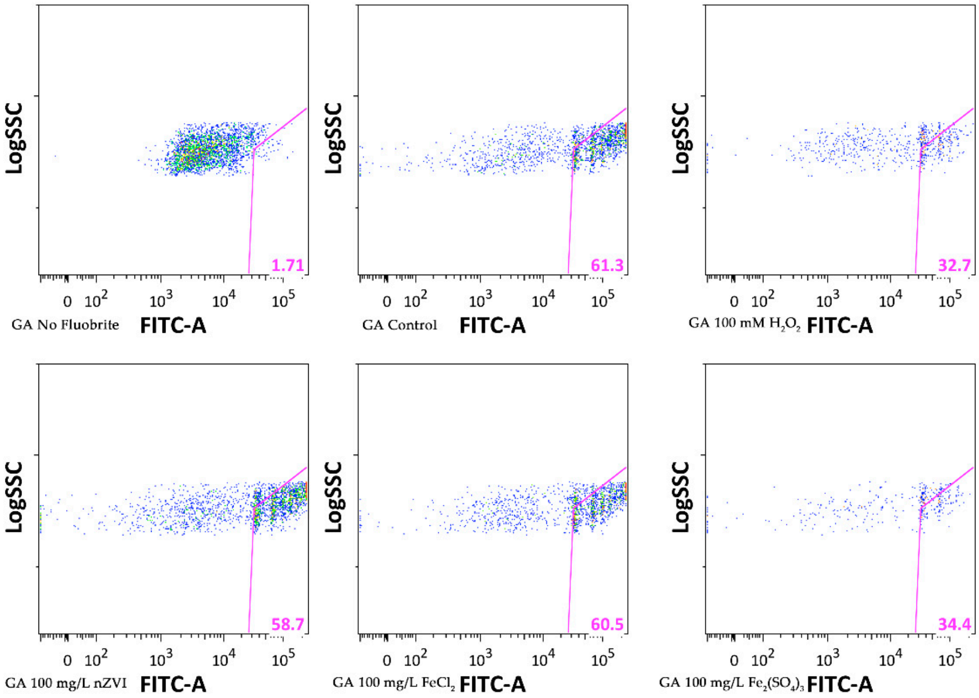

Figure S19. Phagocytic activity of granular amoebocytes (GA) without Fluoresbrite, without treatment (control) and GA exposed to 100 mM H_2O_2 (positive control), 100 mg/L of nZVI, $FeCl_2$ and $Fe_2(SO_4)_3$ after 24 hours.
